# Powerful partnership: crosstalk between pannexin 1 and the cytoskeleton

**DOI:** 10.3389/fphys.2014.00027

**Published:** 2014-01-30

**Authors:** Andrew K. J. Boyce, Leigh E. Wicki-Stordeur, Leigh Anne Swayne

**Affiliations:** ^1^Division of Medical Sciences, University of VictoriaVictoria, BC, Canada; ^2^Department of Biology, University of VictoriaVictoria, BC, Canada; ^3^Department of Biochemistry and Microbiology, University of VictoriaVictoria, BC, Canada; ^4^Department of Cellular and Physiological Sciences and Island Medical Program, University of British ColumbiaVancouver, BC, Canada

**Keywords:** pannexin, Panx1, cytoskeleton, actin, mechanosensitive channels, mechanotransduction

## Abstract

The roles of pannexin 1 (Panx1) large-pore ion and metabolite channels are becoming recognized in many physiological and pathophysiological scenarios. Recent evidence has tightly linked Panx1 trafficking and function to the cytoskeleton, a multi-component network that provides critical structural support, transportation, and scaffolding functions in all cell types. Here we review early work demonstrating the mechanosensitive activation of Panx1 channels, and expand on more recent evidence directly linking Panx1 to the cytoskeleton. Further, we examine the reciprocal regulation between Panx1 and the cytoskeleton, and discuss the involvement of Panx1 in cytoskeletal-regulated cell behaviors. Finally, we identify important gaps in the current knowledge surrounding this emerging Panx1-cytoskeleton relationship.

## Introduction

Mechanical forces shape virtually all biological processes in myriad ways (for a recent review see Lim et al. 2010). The cytoskeleton is a complex interconnected protein meshwork that plays a critical role in cellular biomechanics. Its many components and accessory/regulatory proteins provide structural stability and shape, conduits for the transport of vesicles and macromolecules, and scaffolding for receptors and ion channels. It also communicates with multiple signaling pathways within and outside of cells to modulate these activities in response to the ever-changing demands of cells and their environments (Jaqaman and Grinstein, 2012). *Mechanosensitive channels* provide an important means of crosstalk between chemical and mechanical signaling systems. These are channels that pass molecules and/or ions in response to stretch, and are often intimately associated with the cytoskeleton (reviewed in Hamill 2006).

The pannexins (Panxs) were initially discovered as homologs to the innexin invertebrate gap junction protein family (Panchin et al., 2000). The initial electrophysiological characterization of Panx1 channels provided evidence of a large conductance activated by membrane depolarization (Bruzzone et al., 2003). Soon after this ground-breaking finding, Bao et al. (2004) made a further striking discovery. They uncovered an activation mechanism relating the activation of Panx1 to mechanical deformation, and they provided the first demonstration that Panx1 can form single membrane mechanosensitive channels. They also provided the first evidence for the role of Panx1 in adenosine triphosphate (ATP) release, which is perhaps one of the most well-known features of these large pore channels.

These expression system findings have since been expanded to erythrocytes (Locovei et al., 2006), lung epithelium (Seminario-Vidal et al., 2011; Richter et al., 2014), and more recently, neurons (Xia et al., 2012). Further, Panx1 has been shown to physically interact with the actin cytoskeleton (Bhalla-Gehi et al., 2010; Wicki-Stordeur and Swayne, 2013) and the expression of Panx1 exhibits a significant level of control over multiple cytoskeletal elements (Penuela et al., 2012). Here we discuss these findings and identify key knowledge gaps that will be important to further unravel the potentially powerful relationship between Panx1 and the cytoskeleton.

## Activation of Panx1 by mechanical stress

The first demonstration of stretch-mediated Panx1 opening resulted from work in an ectopic expression system by Bao et al. (2004). The authors investigated whether Panx1 exhibits the properties of a mechanical conduit for ATP by expressing human Panx1 in *Xenopus* oocytes. In cell-free and cell-attached membrane patches, they observed a large conductance attributed to Panx1 expression that exhibited depolarization-dependent activation associated with ATP release. To test for mechanosensitive properties, they used single channel patch clamp coupled with a negative pressure stimulus (via suction applied to the patch pipette). This mechanical stimulation superseded voltage dependent activation, as it occurred over a wide range of membrane potentials.

A network of actin, known as the cellular “cortex,” forms a tight association with the plasma membrane acting as molecular scaffold for ion channels and receptors (recently reviewed in Salbreux et al. 2012). While it is sometimes assumed that the cytoskeleton is not present in excised membrane patches in electrophysiological experiments, *it is in fact normally present* unless specific measures are taken to disrupt the tight cytoskeleton/membrane association (recently reviewed in Hamill 2006). For example, amongst several groups investigating this intriguing question, by elegantly combining scanning force microscopy with patch-clamping techniques, Sakmann's lab (Horber et al., 1995) confirmed the continued presence of the cytoskeleton in cell-free membrane patches. Further, a recent elegant study has demonstrated that the actin cytoskeleton functions as a “molecular device” in the activation of mechanosensitive channels by both concentrating and conducting the forces required for channel opening (Hayakawa et al., 2008). Although this has not yet been directly tested in the context of Panx1 channels, it is certainly of interest in light of the recent discovery of the Panx1 physical association with actin (Bhalla-Gehi et al., 2010; Wicki-Stordeur and Swayne, 2013).

Although quite unlike one another in many ways, erythrocytes, lung epithelium, and neurons are all linked by their responsiveness to mechanical deformation through Panx1-mediated ATP release. Locovei et al. (2006) observed that Panx1 is present in human erythrocytes, and mediates ATP release and ion flux in response to depolarization and mechanical stretch elicited by pressure in the patch pipette. Another group (Seminario-Vidal et al., 2011) later revealed the role of Panx1 as the ATP conduit responsive to bronchial and tracheal epithelial cell swelling (via hypotonic challenge). Interestingly, their data pointed to a mechanism by which RhoA, a regulator of the actin cytoskeleton in the formation of stress fibers, transduces cell swelling to Panx1 opening. Recently, Richter and colleagues confirmed the role of Panx1 in ATP release from lung epithelial cells in response to stretch. In this case ATP release via Panx1 was elicited by changes in hydrostatic pressure (Richter et al., 2014). An intriguing downstream effect of the hydrostatic pressure-induced ATP release from cells was a concomitant activation of K_*ATP*_ channels. It will be interesting to see whether this functional relationship is relevant to other cell types in which Panx1 and K_*ATP*_ channels are co-expressed. More recently, another group (Xia et al., 2012) confirmed the involvement of Panx1 in mechanical deformation-mediated ATP release of retinal ganglion neurons using both a hypotonic solution paradigm and a special cell-stretching chamber.

While mechanical stretch-mediated ATP release can be a physiological phenomenon for erythrocytes and airway epithelia, it is normally associated with pathophysiology in the context of the nervous system. Here, mechanical stretch via impact-mediated axonal deformation or secondary to swelling is associated with neuronal injury (recently reviewed in Laplaca and Prado 2010). We recently showed, however, that nervous system resident neural stem and progenitor cells, migrating neuroblasts and newborn neurons also express Panx1 (Wicki-Stordeur et al., 2012; Wicki-Stordeur and Swayne, 2013). These cells are normally subject to intense and differing mechanical forces from the time they are born through their journey along the rostral migratory stream under physiologically normal conditions. These forces are elicited through the influence of multiple types of extracellular matrix proteins and various geometrical constraints (reviewed in Barros et al. 2011 and Moore and Sheetz 2011).

## Panx1 directly interacts with the actin cytoskeleton

Bhalla-Gehi and colleagues first demonstrated that ectopically expressed Panx1 interacts with actin (through the C-terminus of Panx1), and that actin microfilaments are critical for Panx1 trafficking to and stability at the plasma membrane (Bhalla-Gehi et al., 2010). Cytochalasin B treatment, an actin filament-destabilizing compound, significantly disrupted the plasma membrane distribution and mobility of Panx1-EGFP in the breast cancer-derived, BICR-M1Rk cell line. In contrast, Panx1-EGFP was insensitive to nocodazole-mediated disruption of microtubules.

Actin and its modulator, actin-related protein 3 (Arp3), were two of several cytoskeletal proteins we recently identified as Panx1-interacting proteins by immunoprecipitation coupled to liquid chromatography and tandem mass spectrometry (LC-MS/MS Wicki-Stordeur and Swayne, 2013). We additionally co-precipitated endogenous Panx1, actin and Arp3, further supporting the idea that these physical interactions occur naturally and are relevant to Panx1 function and signaling. Arp3 closely resembles actin monomers in structure and is part of the seven subunit Arp2/3 actin-modifying complex (reviewed in Firat-Karalar and Welch 2011). In fact, Arp2/3 functions as a nucleation site for new microfilaments, which effectively generates a Y-branched network that allows for actin-mediated mechanical force generation (Mogilner, 2006).

## Panx1 is implicated in cell behaviors reliant on actin remodeling

Using pharmacological tools (probenecid), siRNA-mediated Panx1 knockdown and plasmid-mediated Panx1 overexpression, we further determined that Panx1 has a major influence on neurite outgrowth and cell migration in Neuro-2a cells and ventricular zone neural stem and progenitor cells (Wicki-Stordeur and Swayne, 2013). We found that Panx1 is positively associated with cell migration, whereas it negatively regulates neurite outgrowth. Neurite extension and cell migration are two cellular behaviors that are heavily reliant on complex coordination of both actin and microtubular cytoskeletal dynamics (recently reviewed in Schaefer et al. 2008; Kaverina and Straube 2011; Salbreux et al. 2012).

An earlier study on C6 glioma cells engineered to express Panx1, demonstrated that ectopic Panx1 overtakes control of the actomyosin system to accelerate the compaction of multicellular C6 glioma aggregates (Lai et al., 2007). Furthermore, the authors observed an enhancement of ATP release attributable to Panx1 overexpression, as well as P2X7 receptor modulator sensitivity to the Panx1-mediated changes in cell compaction implicated in the remodeling. In addition to these predictable observations, the presence of Panx1 also had a significant impact on the distribution of the actin cortical network.

Work by several groups had earlier established a connection between P2X receptors and the actin cytoskeleton (Kim et al., 2001; Pubill et al., 2001; Pfeiffer et al., 2004). Essentially, P2X7 receptors were shown to interact with actin (Kim et al., 2001), while other groups demonstrated that ATP activation of P2X receptors promotes actin network restructuring through an actin-modifying complex to alter cellular morphology (Pubill et al., 2001; Pfeiffer et al., 2004). In our proteomic analysis of Panx1 interacting partners (Wicki-Stordeur and Swayne, 2013) we identified actin, Arp3 and other cytoskeletal regulators, and additional proteins, but we did not detect P2X7 receptors. It is not known whether P2X7 receptors are bystanders, or are requisite for the crosstalk between Panx1 and the actin cytoskeleton. Further, whether their involvement is cell-type specific or state-specific (physiological vs. pathophysiological; see Morelli et al. 2003 and Homma et al. 2008) remains to be determined.

## Other effects of Panx1 expression on the cytoskeleton

Not only is it likely that Panx1 functionally interacts with the cytoskeleton, but it can also alter the cytoskeletal proteome, as recently shown by Penuela et al. (2012). This group investigated the role of Panx1 in melanoma tumorigenesis and metastasis, and found that increased Panx1 expression correlated with tumor “aggressiveness.” An shRNA-mediated reduction in Panx1 expression was able to revert the tumor cells to a more melanocytic phenotype (reduced cell migration, increased melanin production and process formation). Using a 2D gel/mass spectrometry approach, the authors identified two important cell structure proteins that were down regulated by the reduction in Panx1, vimentin, an intermediate filament protein, and beta-catenin, an important regulator of cell adhesion. Earlier, Lai and colleagues demonstrated that ectopic expression of Panx1 in C6 glioma cells resulted in a dramatically altered cell morphology (Lai et al., 2007). Panx1 expression led to a flattened morphology quite distinct from the spindle-shaped morphology normally exhibited by these cells. The precise cytoskeletal alterations resulting in this striking change in cell shape were not identified.

These studies along with our recent discovery that modulating Panx1 expression and function has a dramatic impact on neurite outgrowth in Neuro-2a cells and ventricular zone neural stem and progenitor cells (Wicki-Stordeur and Swayne, 2013) suggest that Panx1 is an important cytoskeletal regulator.

## Concluding remarks

It is becomingly increasingly clear that the functional role of Panx1 in cells is closely tied to the cytoskeleton. Panx1 is sensitive to stretch, is involved in cytoskeletal-associated cell behaviors and physically interacts with actin. Further, Panx1 exerts influence on the expression of cytoskeletal proteins and when ectopically expressed, can infiltrate and overtake control of the actin cytoskeleton, even though it is not normally present. This suggests that Panx1 is likely a powerful regulator of the cytoskeleton in cells in which it is endogenously expressed.

We are now working on unraveling the mechanistic details underlying the crosstalk between Panx1, actin, the Arp2/3 complex and the other cytoskeletal elements, including elements associated with microtubular dynamics uncovered by our unbiased proteomic analysis of Panx1 interactors in cells that endogenously express Panx1. By studying these interactions, we hope to gain detailed information on the molecular players that are key to Panx1/cytoskeletal crosstalk. This work will bridge significant knowledge gaps in our understanding of the physiological and pathophysiological roles of Panx1.

## Author contributions

Leigh Anne Swayne conceived of the topic, while Leigh Anne Swayne, Andrew K. J. Boyce, and Leigh E. Wicki-Stordeur co-wrote the body of the manuscript. Andrew K. J. Boyce and Leigh E. Wicki-Stordeur together wrote the abstract. Leigh Anne Swayne, Andrew K. J. Boyce, and Leigh E. Wicki-Stordeur revised the manuscript. All authors approve of the manuscript and its contents.

### Conflict of interest statement

The authors declare that the research was conducted in the absence of any commercial or financial relationships that could be construed as a potential conflict of interest.
